# Electrochemical nanostructuring of (111) oriented GaAs crystals: from porous structures to nanowires

**DOI:** 10.3762/bjnano.11.81

**Published:** 2020-06-29

**Authors:** Elena I Monaico, Eduard V Monaico, Veaceslav V Ursaki, Shashank Honnali, Vitalie Postolache, Karin Leistner, Kornelius Nielsch, Ion M Tiginyanu

**Affiliations:** 1 National Center for Materials Study and Testing, Technical University of Moldova, Chisinau MD-2004, Republic of Moldova; 2 Academy of Sciences of Moldova, Chisinau MD-2001, Republic of Moldova; 3 Leibniz IFW Dresden, Helmholtzstr. 20, 01069 Dresden, Germany

**Keywords:** anodization, crystallographically oriented pores, gallium arsenide (GaAs), nanowires, neutral electrolyte, photocurrent, porous GaAs

## Abstract

A comparative study of the anodization processes occurring at the GaAs(111)A and GaAs(111)B surfaces exposed to electrochemical etching in neutral NaCl and acidic HNO_3_ aqueous electrolytes is performed in galvanostatic and potentiostatic anodization modes. Anodization in NaCl electrolytes was found to result in the formation of porous structures with porosity controlled either by current under the galvanostatic anodization, or by the potential under the potentiostatic anodization. Possibilities to produce multilayer porous structures are demonstrated. At the same time, one-step anodization in a HNO_3_ electrolyte is shown to lead to the formation of GaAs triangular shape nanowires with high aspect ratio (400 nm in diameter and 100 µm in length). The new data are compared to those previously obtained through anodizing GaAs(100) wafers in alkaline KOH electrolyte. An IR photodetector based on the GaAs nanowires is demonstrated.

## Introduction

Electrochemical technology became an established and cost-effective approach for the preparation of porous semiconductor matrices and arrays of nanowires with tailored architecture at the submicrometer scale [1–3]. Semiconductor nanotemplates provide many possibilities for nanofabrication through electrochemically filling the pores with metallic nanostructures such as nanowires or nanotubes, resulting in the production of 2D metallo-semiconductor interpenetrating networks, which are promising for various nanoelectronic, optoelectronic, plasmonic, and nanophotonic applications [4–6]. While the growth of crystallographically oriented and current line oriented pores has been demonstrated in a variety of semiconductors [1–3], to date, only crystallographically oriented pores were observed in GaAs crystalline wafers. This observation is a factor limiting the possibilities for the preparation of various GaAs nanostructures, including nanowires.

Semiconductor nanowires, particularly III–V compound nanowires, show potential for their use as active components in solar cells [7–10], photodetectors [11], light-emitting diodes [12], transistors [13], and other applications. A uniform array of parallel nanowires with diameters of about 50 nm and oriented normally to a InP wafer, i.e., along the crystallographic [100] orientation, was obtained after anodic etching at elevated applied voltages [14]. High-aspect-ratio GaAs pillar arrays with triangular cross section were prepared by combining colloidal crystal templating, anisotropic chemical etching, localized anodic etching, and isotropic anodic oxidation [15–16]. However, this is a complex multistep technology. A more simple and cost-effective technology was applied for obtaining triangular GaAs nanowires through electrochemical etching of GaAs(100) surfaces in aqueous KOH solution [17]. However, this process was difficult to control. The bundles of GaAs nanowires were formed only in some regions of the surface and the orientation of the arrays was basically random.

Usually, acidic or alcaline electrolytes are used for the electrochemical preparation of porous semiconductors although the anodization in environmentally friendly electrolytes, including aqueous solutions of NaCl, has attracted increasing attention during the last decade [5,18–21].

In this paper, we report on the electrochemical porosification of GaAs(111) wafers in neutral NaCl and acidic HNO_3_ electrolytes, and on optimized technological conditions for the one-step formation of GaAs nanowires with triangular shape and well oriented perpendicularly to the substrate.

## Results and Discussion

**Figure 1:**
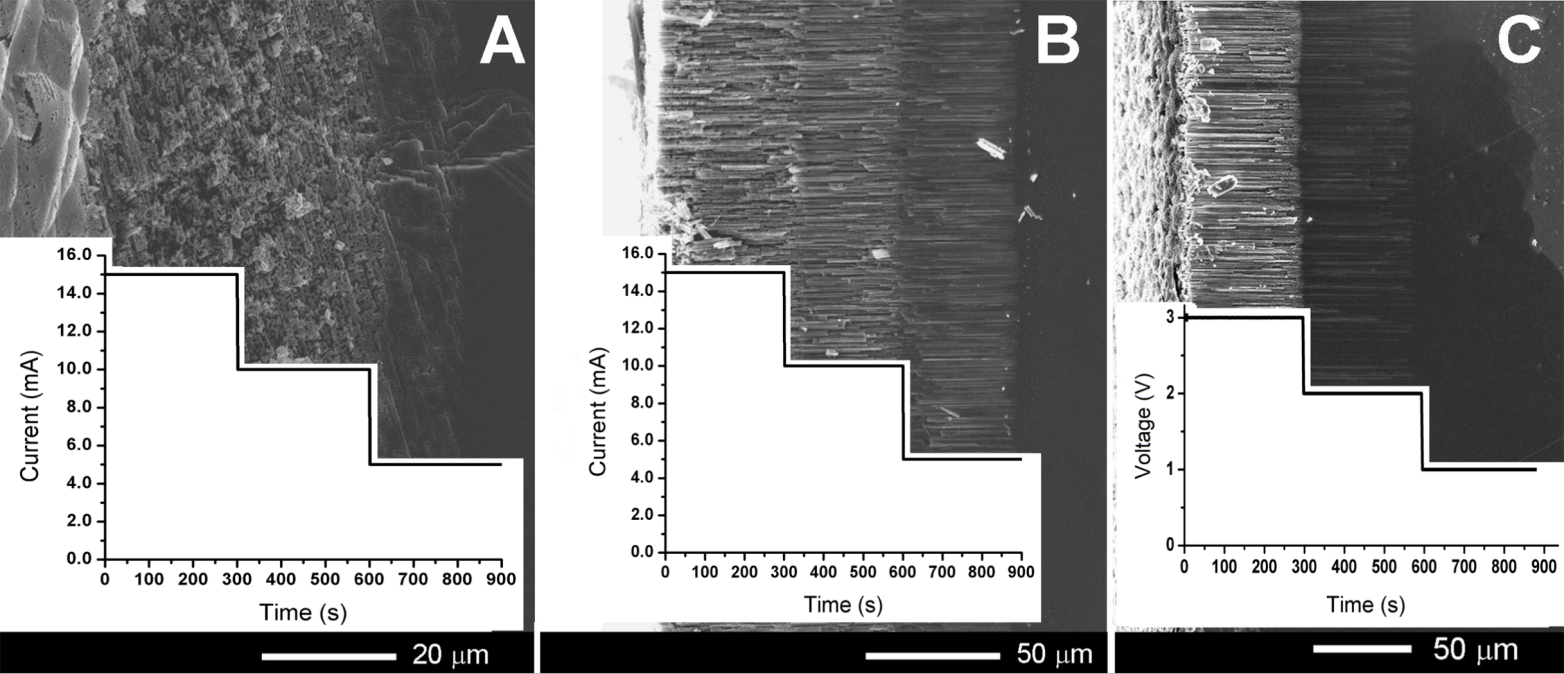
SEM images in cross section of porous GaAs layers for three different conditions of anodization in 1.75 M NaCl: (A) galvanostatic regime on the GaAs(111)A surface; (B) galvanostatic regime on the GaAs(111)B surface; (C) potentiostatic regime on the GaAs(111)B surface. The substrate is on the right side of the images. The inserted plots represent the applied currents (A, B) and the applied potential (C) during anodization.

In order to study the influence of the applied anodization conditions on the porosification process of (111) oriented GaAs crystals, three different current densities and three different voltages were used for galvanostatic and potentiostatic modes, respectively. SEM images of a GaAs crystal anodized on both surfaces in 1.75 M NaCl electrolyte are given in Figure 1. The porous features produced by GaAs anodization in NaCl electrolyte are similar to those previously observed in GaAs samples with the same carrier concentration anodized in HCl electrolyte [1]. This observation corroborates the results obtained from other III–V semiconductors, which state that the etching behavior depends mainly on the anions rather than the cations [22], because the anions of NaCl and HCl electrolytes are the same. The morphology of the porous structure produced by galvanostatic anodization of GaAs(111)A surface (Figure 1A) consists of two sets of ⟨111⟩ crystallographically oriented pores intersecting each other [2]. According to previous studies, the main property of crystallographically oriented pores is their growth along definite crystallographic directions. On substrates with sphalerite crystal structures, the pores grow along the ⟨111⟩B crystallographic directions, regardless of the initial surface orientation, with an angle of approximately 109° between the pores. The pores tend to have a triangular cross section while the pore walls and tips exhibit a pronounced crystallographic anisotropy. A specific characteristic feature of crystallographically oriented pores is their ability to intersect each other without changing their direction of propagation during growth. As one can see from Figure 1A, the degree of porosity decreases with decreasing the anodization current density.

The situation is different when the GaAs crystals are anodized on the (111)B surface. During anodization in the galvanostatic mode at current densities similar to those applied during anodizing the (111)A surface (15, 10, and 5 mA), three porous layers are formed with different degrees of porosity, but the pores are parallel to each other and they grow perpendicularly to the crystal surface (Figure 1B). The same propagation of pores was observed for the potentiostatic mode of anodization (Figure 1C). Switching the anodization voltage to 1 V stops the formation of the porous layer, since this value is below the pore nucleation potential (1.8 V) established from the *I*–*V* characteristic, which is in agreement with the doping level of the used crystal. We will focus further on the results obtained by anodization of the (111)B surface because the resulting morphology is of great interest for the development of porous nanotemplates and elaboration of free-standing nanomembranes based on GaAs.

**Figure 2:**
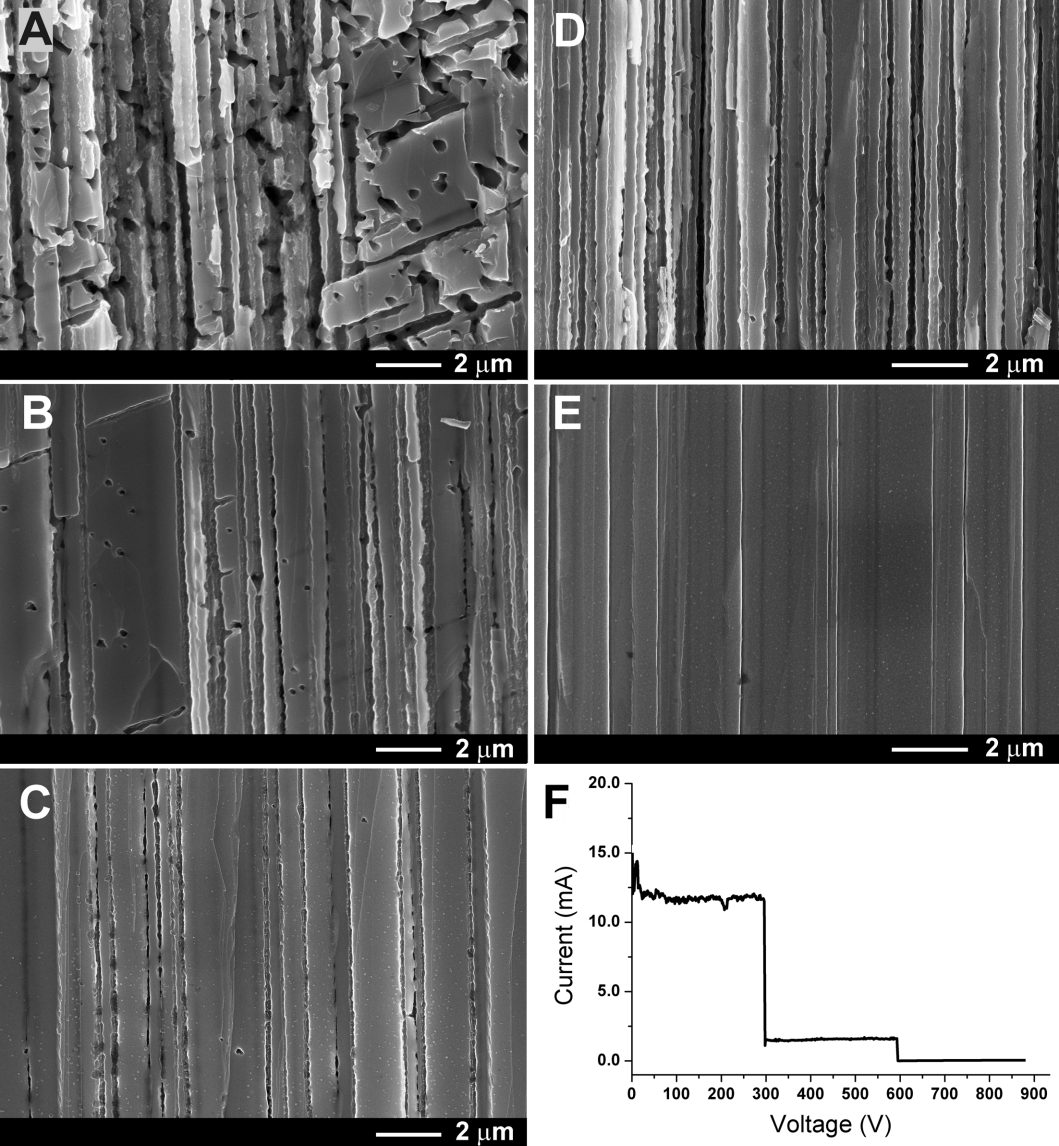
(A–C) SEM images in cross section at higher magnification of the porous layers obtained by anodization of the GaAs(111)B surface in galvanostatic regime, corresponding to the images illustrated in Figure 1B. (D, E) SEM images of samples anodized in potentiostatic regime, corresponding to images illustrated in Figure 1C. (F) Evolution of the current during anodization in potentiostatic regime of the GaAs(111)B surface at applied voltages of 3, 2 and 1 V.

The results of a comparative anodization study of the GaAs(111)B surface in galvanostatic and potentiostatic regimes are illustrated in the SEM images of Figure 2. Along with pores propagating perpendicularly to the substrate surface, pores growing in other directions are observed in the cross-sectional view (Figure 2A), hinting at the formation of pores that are tilted and intersect each other. However, with the decrease of the current density (Figure 2B,C), the number of tilted pores is drastically reduced, and pores oriented perpendicularly to the surface predominate in the produced porous layer (Figure 2C). The morphology of the layer produced by anodization in the potentiostatic regime presented in Figure 2D,E looks more uniform (note that the pores grow perpendicularly to the surface). It should be mentioned, however, that the pore walls are not smooth, which is observed for both potentiostatic and galvanostatic anodization regimes. This can be explained by analyzing the current as a function of the time at constant voltage (Figure 2F). At the applied potential of 3 V, the registered current is practically stable at a value of 13 mA, but some fluctuations in the current can be easily observed. Reducing the anodization voltage to 2 V leads to a decrease of the current down to 5 mA. This value remains quite stable. So, by anodizing the sample in the potentiostatic mode at relatively low voltage, which does not introduce self-oscillations in the registered current, it is possible to obtain pores with smooth walls (Figure 2E).

**Figure 3:**
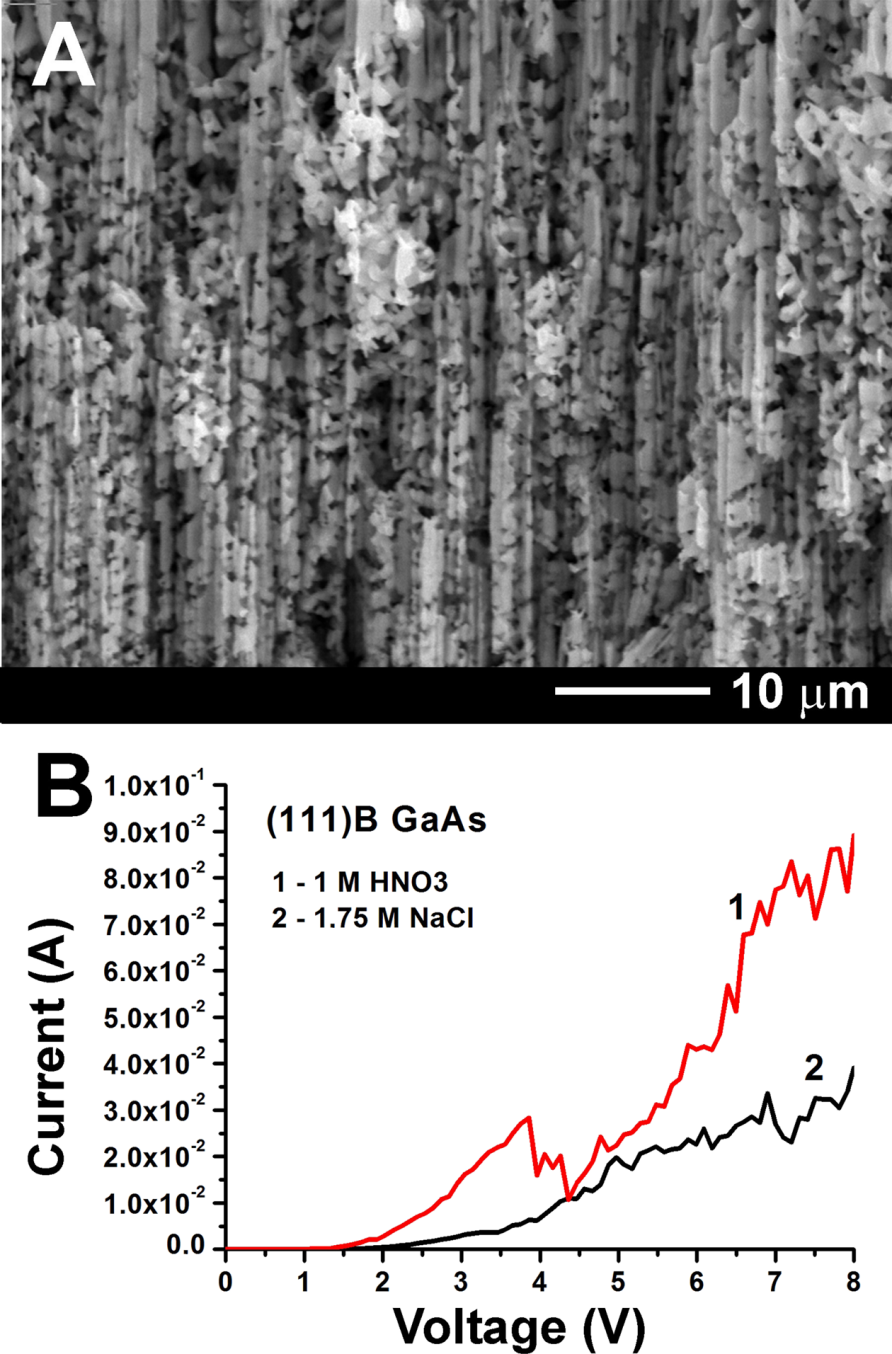
(A) SEM images of the formation of interrupted GaAs nanowires on the (111)B surface anodized in NaCl electrolyte at 6 V. (B) The polarization curves measured at a scan rate of 50 mV·s^−1^ at the beginning of the anodization process of a GaAs(111)B crystal in 1 M HNO_3_ (curve 1) and 1.75 M NaCl (curve 2).

According to Li and co-workers [17], the anodization of GaAs with a carrier concentration of about 10^18^ cm^−3^ in a KOH electrolyte can be categorized into three etching modes, deduced from the analysis of the current–voltage plot at a scan rate of 5 mV·s^−1^. Practically no etching occurs when the applied voltage is below 3 V, which is the pore formation potential. The first etching mode is characterized by the formation of triangular nanowires, which occurs at an applied potential in the range of 3.0–4.5 V. The second mode corresponds to surface texturing occurring at an applied potential in the range of 4.5–6.5 V. At an applied potential higher than 6.5 V the sample is electropolished. Taking these observations into account, we increased the applied potential in our experiments to 4 V, with the purpose of producing GaAs nanowires. However, the analysis of SEM images after anodization in NaCl electrolyte with an applied potential of 4 V revealed that no nanowires are formed. Instead a higher number of tilted pores was formed, similar to the morphology produced under anodization in the galvanostatic regime with a current of 14 mA (Figure 2A). Moreover, an increase of the applied potential to 6 V leads to the formation of GaAs nanowires, but the number of tilted pores that intersect the vertical nanowires increases, resulting in the formation of interrupted nanowires (Figure 3A). A further increase of the applied voltage is not recommended, because the formed nanowires are destroyed. We are interested in the fabrication of pores strictly perpendicular to the crystal surface, which would enable us to fabricate stable nanowires.

One can conclude that anodizations in NaCl electrolyte and in aqueous HCl solution are suitable for the production of porous layers with various morphologies, rather than for the fabrication of GaAs nanowires. Since, on one hand, nanowires were produced through anodization in alkaline KOH electrolyte [17] and, on the other hand, the etching behavior in acidic electrolytes is mainly determined by the anions [22], we tried to obtain GaAs nanowires through anodization in acidic electrolyte with another type of anion. A comparison of the polarization curves measured at the beginning of the anodization process of a GaAs(111)B crystal in 1 M HNO_3_ or 1.75 M NaCl electrolyte is presented in Figure 3B. It can be observed that current oscillations occur at applied potentials higher than 4 V for both electrolytes. We suppose that these self-induced oscillations are related to the simultaneous formation of pores along the ⟨111⟩B directions and an increasing contribution of tilted pores. At high anodizing voltages electropolishing occurs in both electrolytes. The potential for initiating electropolishing is 4.5 V and 6 V for anodization in HNO_3_ and NaCl electrolyte, respectively. Note that the value for anodizing in NaCl electrolyte agrees with previously reported data [17].

**Figure 4:**
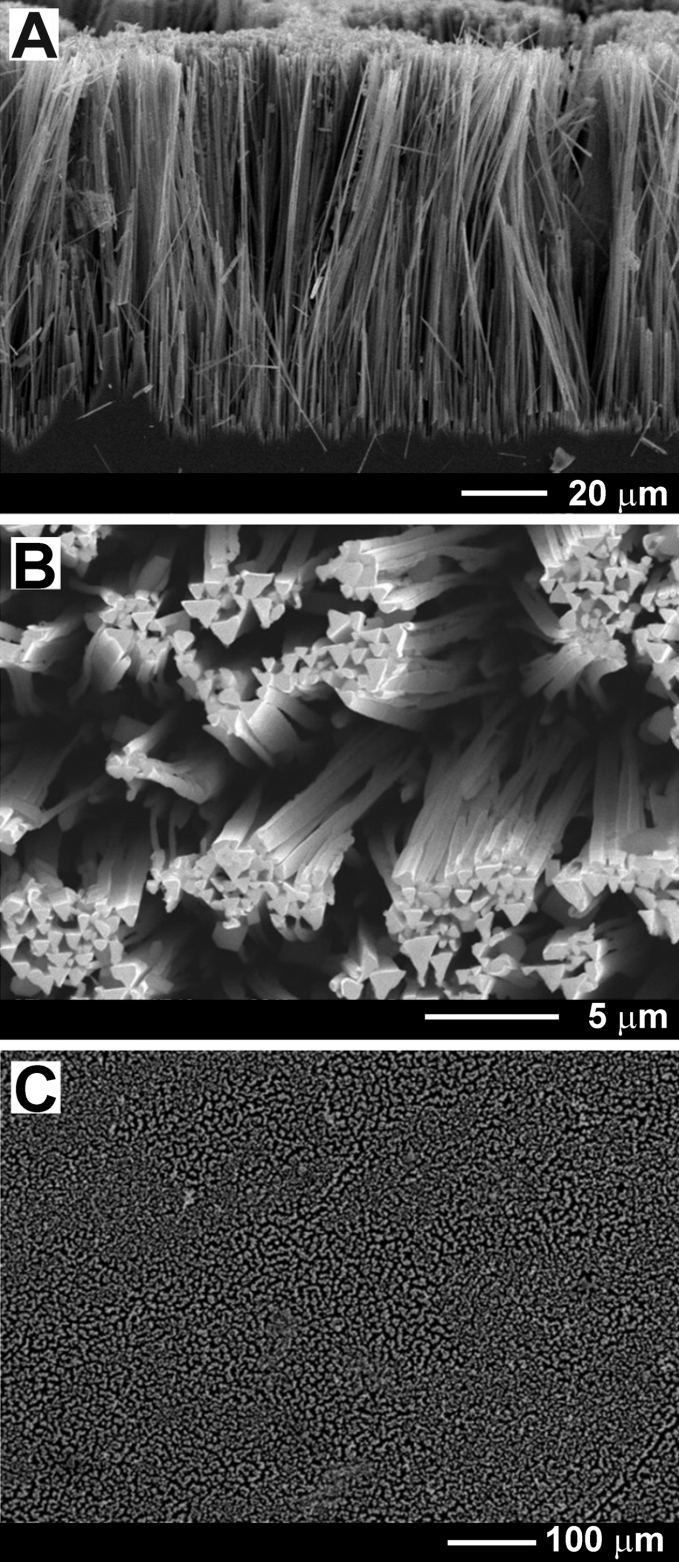
(A) SEM image in cross section of a GaAs(111)B sample anodized at 3 V for 20 min in 1 M HNO_3_. (B, C) Top-view SEM images.

Arrays of GaAs nanowires with a very high aspect ratio and well oriented perpendicularly to the (111)B crystal surface were produced under anodization at an applied potential of 3 V for 20 min in 1 M HNO_3_ electrolyte (Figure 4A). The obtained triangular nanowires with a diameter of 400 nm are 100 µm in length, i.e., the aspect ratio is around 250. The uniform distribution of the nanowires across the sample surface is illustrated in Figure 4C. Interestingly, the anodization potential for producing nanowires in the acidic 1 M HNO_3_ electrolyte is similar to that required for producing GaAs nanowires in alkaline 5% KOH electrolyte [17].

Thus, triangular-shape GaAs nanowires with a diameter ranging from 200 to 400 nm can be produced by electrochemical etching of GaAs(100) wafers with a carrier concentration of the order of 10^18^ cm^−3^ in KOH electrolyte, or by etching of GaAs(111)B substrates in HNO_3_ electrolyte. However, the bundles of GaAs nanowires are formed only in some regions of the surface anodized in KOH electrolyte, and the orientation of the arrays is basically random, while the nanowires produced in HNO_3_ electrolyte are highly oriented perpendicularly to the wafer surface. No nanowires were reported after GaAs anodization in H_2_SO_4_ electrolyte, which was used for uniform pore nucleation in a two-step anodization process and the preparation of porous structures consisting of crossing pores [1,23]. GaAs nanocolumns with a diameter of about 200 nm were obtained previously through anodization of GaAs(100) substrates in aqueous HCl solution [24], but the nanocolumns were penetrated by a large number of tilted pores. One can conclude that etching in HNO_3_ electrolyte suppresses the nucleation of tilted pores as compared to anodization in HCl or H_2_SO_4_ electrolyte, resulting in the fabrication of non-porous GaAs nanowires, which are better suitable for photodetector applications. High-aspect-ratio arrays of spatially ordered triangular GaAs nanopillars with diameters of about 1.5 µm were previously fabricated by a combination of electrochemical etching in HCl electrolyte of a pre-etched GaAs(111) substrate and anisotropic chemical etching [15–16]. However, this is a multistep process requiring the use of photolithography with colloidal crystal templating in the first pre-etching step. Fabrication of GaAs nanopillars by metal-assisted chemical etching (MacEtch) also requires lithographic methods for catalytic metal patterning [25–26]. Among non-lithographic methods based on electrochemical etching, to the best of our knowledge, there is only one report on the preparation of high-aspect-ratio vertically aligned GaAs nanowires with a diameter of about 200 nm and a length of 100 µm on GaAs(111)B wafers with a carrier concentration in the range of (1–2) × 10^18^ cm^−3^ [27]. Bundles of these nanowires were tested for field emission, but not for photodetector applications. The nanowires were produced using electrochemical etching under optimized conditions in a mixed H_3_PO_4_/HCl electrolyte followed by chemical etching for reducing the diameter down to 150 nm. The authors of the work did not discuss the diameter distribution of nanowires produced by the process, effectively utilizing a structural feature of spontaneously generated patterns. According to the presented scanning electron microscope images, however, there is a relatively broad distribution.

The nanowires produced in the present work also exhibit a diameter distribution with a certain width. However, we believe that the distribution could be narrowed by further optimization of the technological processes. Note that the diameter of a particular nanowire is determined by the width of the space charge region (SCR) set up within the nanopore wall at the interface with the electrolyte during anodization [23], and it equals ca. 2 × SCR [17]. As a result, for higher carrier concentrations in the GaAs wafer thinner nanowires are obtained. The density of the nanowires is quite homogeneous on a large scale (Figure 4C), while the nanowires tend to form bundles at the micrometer scale, similar to the results presented in [27].

**Figure 5:**
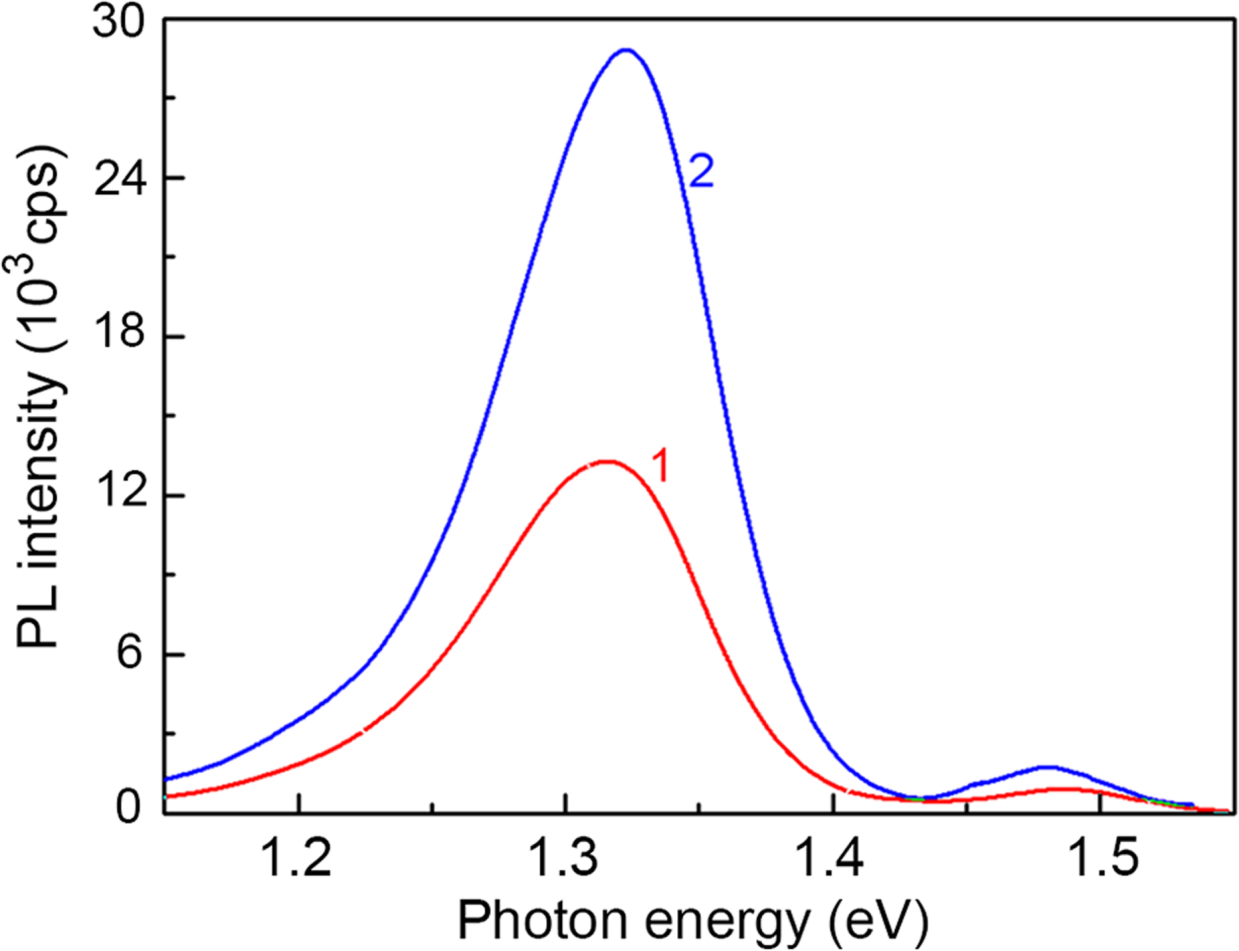
PL spectra of bulk (curve 1) and anodized (curve 2) GaAs samples measured at a temperature of 10 K.

Figure 5 compares the photoluminescence (PL) spectra of the as-grown and anodized GaAs samples measured at low temperature. One can see that the shapes of the spectra are similar. The only difference is the emission intensity, which is higher by a factor of about two in the anodized GaAs sample. This means that the PL spectra can be attributed to identical recombination channels. In both samples the PL is dominated by an emission band around 1.32 eV with a weaker PL band at 1.485 eV. The PL band at 1.32 eV is usually attributed to Si impurities at the Ga sites forming different complexes, such as (Si_Ga_V_Ga_) [28–29] or (Si_Ga_Ga_As_) [30]. Since Si impurities exhibit an amphoteric behavior in GaAs, they give rise to an acceptor Si_As_ state in addition to the Si_Ga_ shallow donor state. The second PL band at 1.485 eV is related to the recombination of electrons from the conduction band with holes trapped by the Si_As_ state the energy level of which is situated 35 meV above the valence band [31]. The higher intensity of the emission from the anodized GaAs sample is indicative of an effective passivation during anodization of the huge internal surface of the porous sample [18].

**Figure 6:**
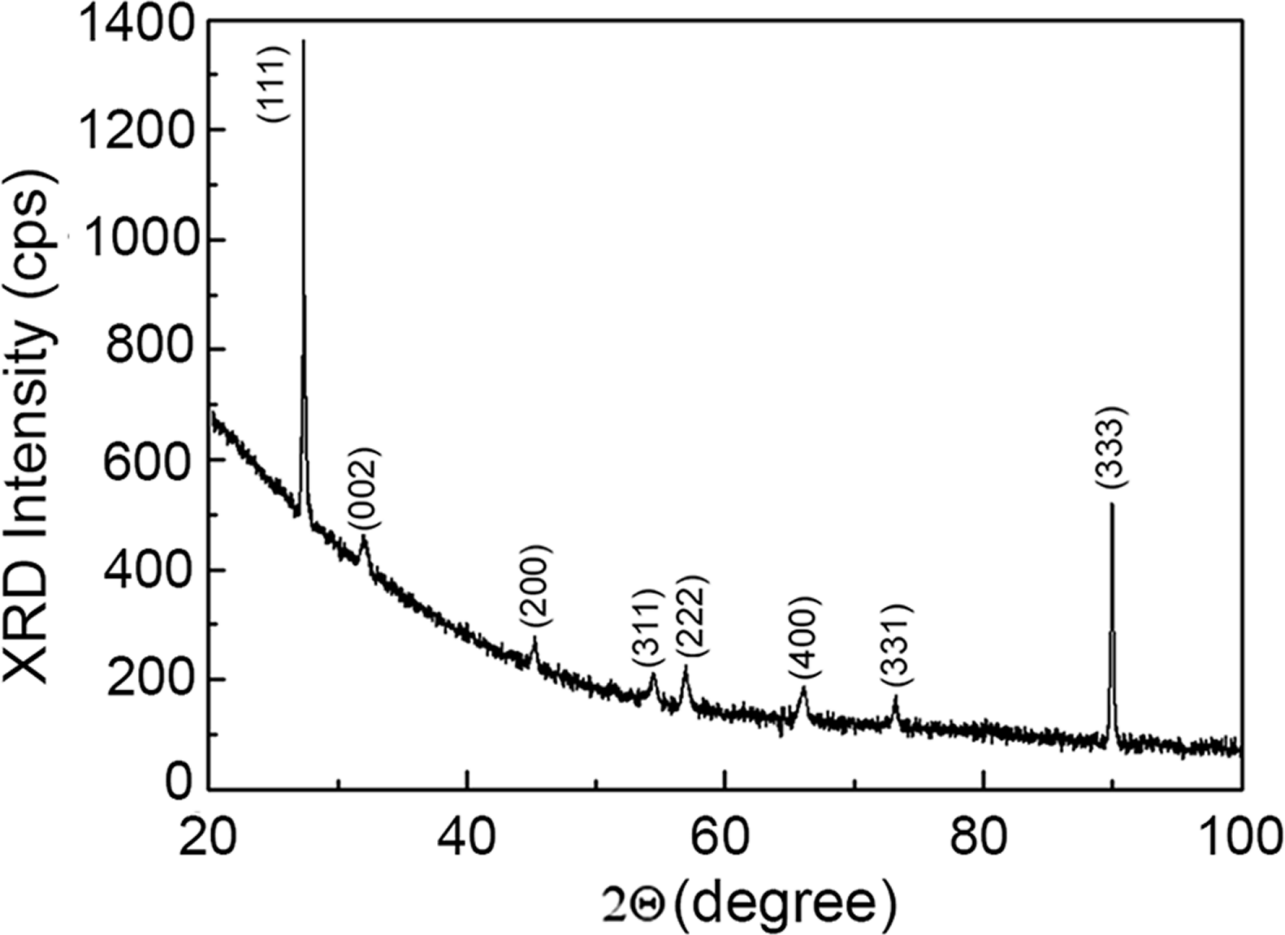
XRD pattern of the anodized GaAs(111)B sample.

The preservation of the quality of the GaAs compound after anodization is also confirmed by the results of XRD analysis (Figure 6). The high quality of the material produced by anodization is indicated by narrow reflexes with a full width at half maximum (FWHM) of about 0.08°. The predominance of (111) and (333) reflexes in the XRD pattern indicates also to the preservation of the initial (111)B crystallographic orientation of the sample.

**Figure 7:**
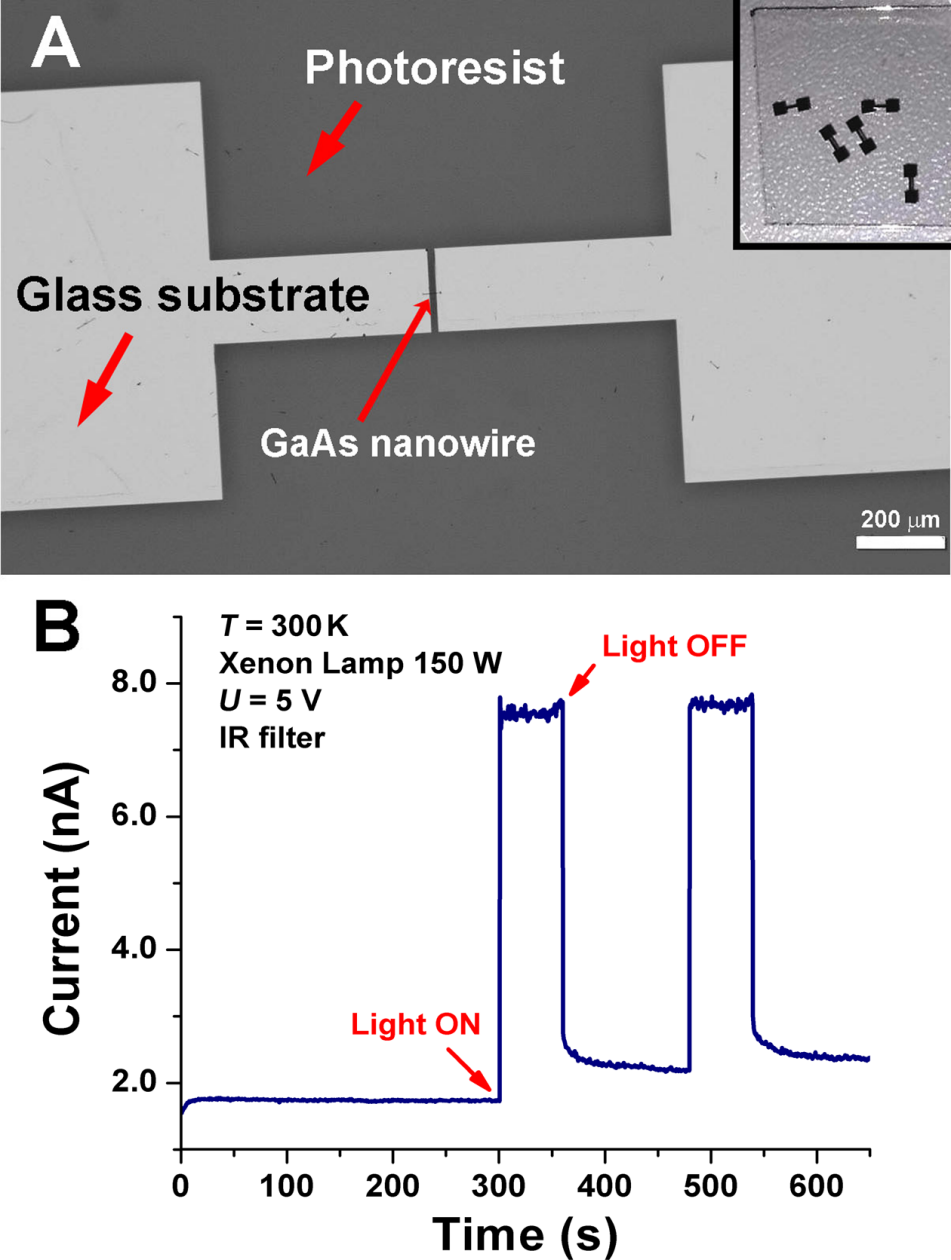
(A) Optical microscopy image of the opened regions in the photoresist on the glass substrate for deposition of the metal contacts on the selected GaAs nanowire. The inset in (A) shows a photo of five contacted GaAs nanowires on the same glass substrate. (B) Photocurrent build-up and relaxation of the photodetector measured for an IR illumination density of 800 mW·cm^−2^.

To demonstrate the applicability of the nanowires for device fabrication, a photodetector for the IR region of the spectrum was tested, as described in the Experimental section. A special design of contacts was applied via laser beam lithography on selected nanowires. As illustrated in Figure 7A, defined regions (bright regions) were opened in the photoresist (dark regions) for further metal deposition. Note that the nanowire was visible in the optical microscope due to its length of 70 µm, despite the small diameter. The distance between the contacts is 20 µm. A photograph of five contacted nanowires on a glass substrate after Cr/Au deposition and lift-off is presented in the inset of the Figure 7A. The photocurrent build-up and relaxation for a photodetector produced on a nanowire with a diameter of 400 nm is presented in Figure 7B for an IR illumination density of 800 mW·cm^−2^. One can see that the current increases by a factor of four in magnitude under illumination with IR light.

**Figure 8:**
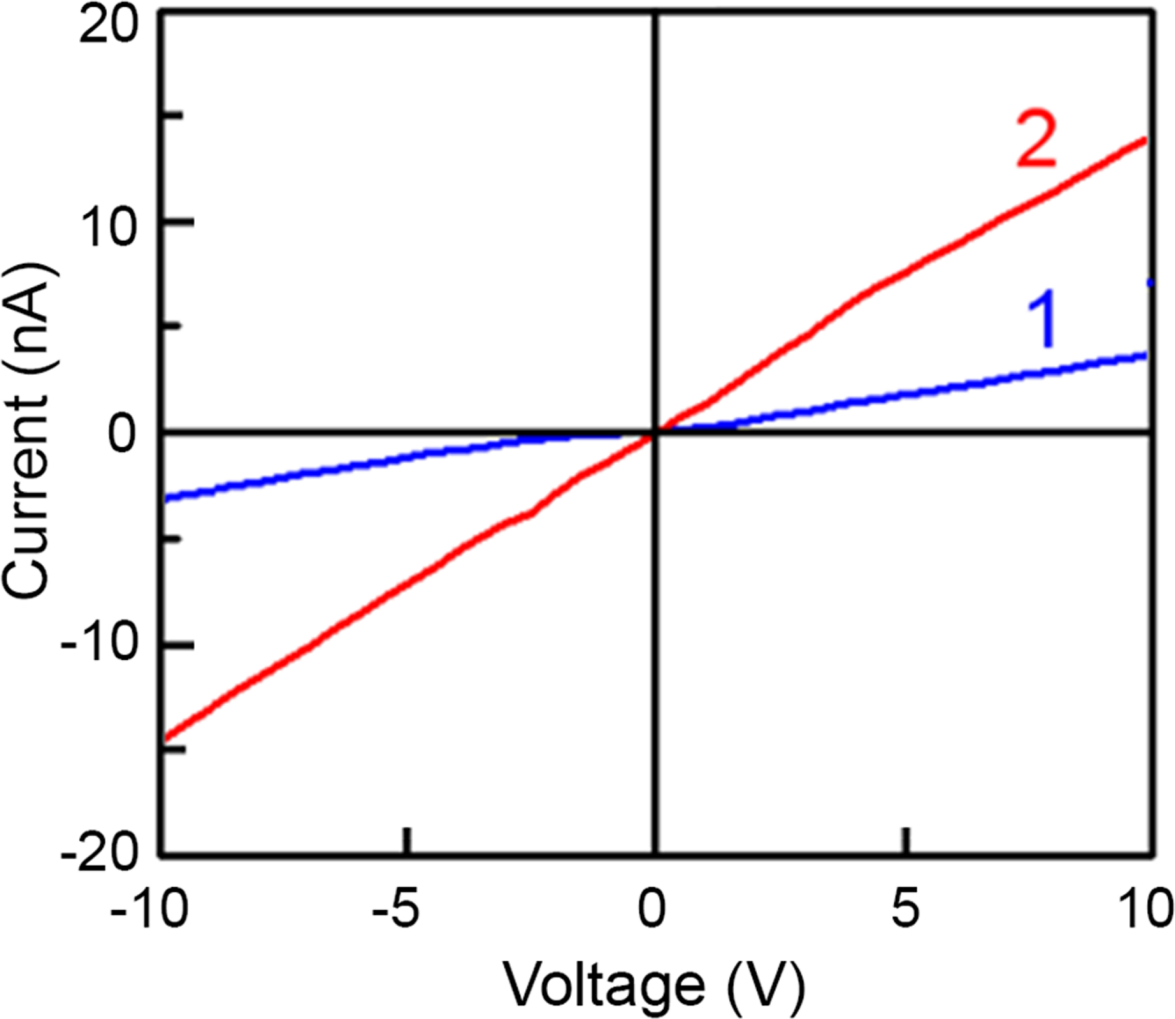
Current–voltage characteristics measured in dark (curve 1) and under IR illumination with power density of 800 mW·cm^−2^ (curve 2) for the GaAs nanowire photodetector with the design shown in Figure 7.

The current–voltage characteristics measured with and without illumination reveal a good ohmic quality of the prepared Cr/Au contacts (Figure 8). Therefore, one can conclude that the detector works in the photoconductor mode.

The responsivity of the detector is defined as

[1] $$R=\frac{I_{\text{photo}}-I_{\text{dark}}}{P_{\text{ill}}},$$

where *I*_photo_ is the photocurrent of the photodetector, *I*_dark_ is the dark current, and *P*_ill_ is the illumination power on the photodetector. The calculated responsivity of the GaAs nanowire photodetector equals 100 mA·W^−1^, according to the data presented in Figure 7B for a nanowire with a diameter of 400 nm and a length of 20 µm biased at 5 V.

The estimation of the detectivity D*, which is defined as

[2] $$D^{*}=\frac{R\sqrt{A}}{\sqrt{2eI_{\text{dark}}}},$$

where *A* is the active area of the photodetector, and *e* is the elementary charge, gives a value of ca. 1.2 × 10^9^ cm·Hz^1/2^·W^−1^, under the assumption that shot noise is the primary source of noise in the detector [32].

Taking into account that the photodetector works in the photoconductor mode, the photocurrent increases linearly with increasing bias. This results in increasing responsivity and detectivity with increasing bias. For instance, the responsivity increases by a factor of three after increasing the bias from 5 to 20 V. The measured parameters vary among the five investigated devices basically due to the different diameters of the nanowires. The responsivity measured at the same excitation power density of 800 mW·cm^−2^ at a bias of 5 V decreases with decreasing nanowire diameter. The detectivity also decreases with decreasing diameter, but to a lesser extent.

The obtained values of responsivity and detectivity are comparable with those previously reported for a graphene/GaAs NW photodetector with a Schottky junction working at 532 nm radiation [33]. The detectivity of our photodetector is better than that reported for a GaAsSb NW IR detector (1300 nm), although the responsivity of the GaAsSb NW detector is better [34]. A photodetector based on a single GaAs nanowire with a responsivity of 1.2 mA·W^−1^ has been recently reported on a nanowire prepared by chemical beam epitaxy (CBE) with a vapor–liquid–solid (VLS) growth procedure [35]. This value is by two orders of magnitude lower than the responsivity of our photodetector. However, one should take into account that it was fabricated with a nanowire that is one order of magnitude thinner than our nanowire prepared by electrochemical etching. The detectivity of our GaAs nanowire detector working in the photoconductor mode is by a factor of 1.5 better than the value obtained recently on molecular beam epitaxy (MBE)-grown Si-doped GaAs nanowires with a carrier concentration of 1.47 × 10^17^ cm^−3^, working in the field-effect transistor (FET) mode at similar excitation power densities (around 800 mW·cm^−2^) but with radiation of 532 nm wavelength [36]. At the same time, the authors of [36] succeeded to improve the detectivity of the NW FET detector by one order of magnitude and also to attain record responsivities of the order of 1 kA·W^−1^ after optimization of the carrier concentration in the GaAs NWs and of the photodetector design. We suppose that the parameters of IR photodetectors based on nanowires prepared by anodization can also be significantly improved after corresponding optimization.

A drawback of photoconductive detectors based on semiconductor nanowires is related to their long-relaxation phenomena caused by the strong surface band bending effects [37]. In contrast, much shorter relaxation times are inherent to photodetectors based on interdigitated metal–semiconductor–metal structures with Schottky diodes. However, a very low feature size is needed for such structures, which makes photolithography challenging [38].

## Conclusion

This study demonstrates possibilities to produce porous GaAs structures with a controlled degree of porosity through the anodization of GaAs(111) wafers in a neutral, environmentally friendly NaCl electrolyte. Porous morphologies with pores oriented perpendicularly to the wafer surface are obtained through potentiostatic anodization of GaAs(111)B surfaces at low applied potentials. With increasing the applied potential in the potentiostatic anodization mode, or the current in the galvanostatic anodizing mode, a higher number of tilted pores are produced in addition to those oriented perpendicularly to the wafer surface. When the anodization is performed on the GaAs(111)A surface, a porous morphology with crossing pores is obtained, and the degree of porosity increases with increasing the anodizing current. Since these results are similar to those previously observed after anodization of GaAs wafers in HCl electrolytes, one can conclude that the etching behavior is mainly determined by the type of anions. No nanowires are produced under any anodizing conditions in NaCl electrolyte. On the other hand, high-aspect-ratio triangular shape GaAs nanowires are obtained by anodizing in a HNO_3_ electrolyte at an applied potential of 3 V, and the uniformity and orientation of these nanowire arrays are much better than those produced previously with anodizing in alkaline KOH electrolytes. The produced GaAs nanowires prove to be suitable for the development of IR photodetectors with good sensitivity and dynamic characteristics.

## Experimental

**Electrochemical anodization.** Crystalline 500 µm thick (111)-oriented substrates of Si-doped n-GaAs with a free electron concentration of 2 × 10^18^ cm^−3^ were used in this study. The samples were sonicated in acetone for 15 min, cleaned in distilled water and dried. In order to remove the native oxide from the surface, the samples were dipped in a HCl/H_2_O (1:3) solution for 2 min. The electrical contacts to the sample were prepared with silver paste, then the samples were pressed against an O-ring in a Teflon cell with the 0.2 cm^2^ area exposed to the electrolyte. The electrolytes used in this study were 1.75 M NaCl and 1 M HNO_3_. The experiments were performed in a three-electrode configuration, with a Pt mesh with a surface area of 6 cm^2^ acting as counter electrode, a saturated Ag/AgCl reference electrode and the sample as working electrode. The anodization was performed in galvanostatic as well as potentiostatic regimes at room temperature (*T* = 23 °C). Analysis of morphology and chemical composition of the anodized GaAs crystals was carried out using scanning electron microscopy (Zeiss Sigma and TESCAN Vega TS 5130 MM equipped with an Oxford Instruments INCA Energy EDX system operated at 20 kV). The photoluminescence spectra were measured with a double spectrometer with resolution better than 1 meV under excitation by the 514 nm line of an Ar^+^ SpectraPhysics laser. The samples were mounted on the cold station of a LTS-22-C-330 cryogenic system. X-ray diffraction analysis of the samples was performed with a Philips X-Pert MPD System with Cu Kα_1_ radiation.

**Electrical contacts to GaAs nanowires.** The contacts were realized using laser leam lithography (µPG 101, Heidelberg Instruments). After the formation of nanowires via anodization, the GaAs substrates were treated in an ultrasound bath for 15 s in ethanol. Subsequently, a few drops of the ethanol suspension containing nanowires were deposited on a glass substrate followed by a gentle blow drying to remove the ethanol. A double-layer resist (LOR 3B and ma-P 1205) was spin-coated on the glass substrate with the GaAs nanowires and was exposed with the pattern containing the contact pad structure of 1.5 mm × 1.5 mm using the laser writer. After the development of the exposed contact pad structure, a thin layer of 50 nm Cr followed by 250 nm Au layer was sputtered using a magnetron from Torr International Inc model No: CRC622-2G2-RF-DC and lift-off was performed with Microposit remover 1165 at 50 °C.

**Photoelectrical characterization.** To excite photoconductivity in the GaAs nanowires, the radiation from a Xenon lamp DKSS-150 was used. An optical filter was used to select radiation from the near-IR spectral range (700–2500 nm, optical power 130 mW). The current through the samples was measured by means of a Keithley’s Series 2400 source measure unit. Since the photoconductivity decay time is long enough, a mechanical shutter was used in the relaxation experiments. The signal from the source measure unit was fed to computer via IEEE-488 interface for further data processing. The measurements were performed at 300 K.
